# WDR5 is a prognostic biomarker of brain metastasis from non-small cell lung cancer

**DOI:** 10.3389/fonc.2022.1023776

**Published:** 2022-09-29

**Authors:** Zheng Li, Nan Liang, Na Wang, Yan Jia, Cui Tian

**Affiliations:** ^1^ Department of Neurosurgery, The Second Affiliated Hospital of Shandong First Medical University, Tai’an, China; ^2^ Department of Anesthesiology, The First People’s Hospital of Tai’an, Tai’an, China; ^3^ Department of Intensive Care Unit, Shandong Provincial Tai’shan Hospital, Tai’an, China; ^4^ Department of Intensive Care Unit, The Second Affiliated Hospital of Shandong First Medical University, Tai’an, China

**Keywords:** WDR5, lung cancer, brain metastasis, prognosis, biomarker, patient cohort

## Abstract

**Background:**

Lung cancer (LC) is the most frequent caner type and causes the most cancer-related death. Brain metastases (BM) are the deadliest complications of lung cancer, and the prognostic biomarkers of BM are urgently needed.

**Materials and methods:**

In our study, we established an inception cohort including 122 patients with asynchronous BM from NSCLC, and further selected 70 patients who received surgical resection, which compromised the validation cohort. With immunohistochemistry, we investigated the expression of WDR5 in the cohort. By chi-square method, the correlations between WDR5 and clinicopathological factors were analyzed. The prognostic indicators were analyzed with the univariate analysis, and independent prognostic factors were identified by multivariate analysis with Cox-regression model.

**Results:**

WDR5 is frequently expressed in the cytoplasm of BM from NSCLC. Patients with low or high expression of WDR5 account for 60% and 40% respectively. High expression of WDR5 indicates poor prognosis of BM from NSCLC (*P*=0.001). In addition to WDR5, KPS is also a prognostic factor of BM, and high KPS predicts favorable prognosis (*P*=0.006). WDR5 is an independent prognostic biomarker for poor prognosis of BM from NSCLC, with the cancer-related odds as 2.48.

**Conclusions:**

High expression of WDR5 can predict the poor prognosis of BM, and WDR5 is an independent prognostic biomarker of BM from NSCLC. Patients with WDR5 overexpression are more high-risk to suffer BM-related death and should receive more intense post-operational supervision.

## Introduction

Lung cancer (LC) is the malignancy with the highest morbidity and it results in most cancer-related death worldwide, accounting for 18.4% of all cancer-related deaths (1). Approximately 2.1 million patients were diagnosed as lung cancer in 2018 (1). The public health burden of LC is much more challenging in developing countries such as China, because they usually have more severe air pollution and higher LC frequencies (2). Histologically, LC can be divided into non-small cell lung cancer (NSCLC) and small cell lung cancer (SCLC) (3). NSCLC accounts for approximately 85% of all LCs, which can be further categorized into adenocarcinoma (LAD), squamous cell (SQCC), and large cell carcinoma (LCC) (4, 5).

Brain metastases (BM) are the most common and also the most devastating complications for the patients with LC, taking up approximately 30% of patients with advanced-stage LC (6). Every year, about 150,000 cancer patients develop BM in the United States (7), and LC is the main cause of secondary BM (8, 9). The median survival time of BM from NSCLC ranges from 3 to 14.8 months (10). The effect of chemotherapy is very limited because of blood brain barrier, and surgical resection is one of the treatment options, especially to those who have the opportunity of surgical resection or have significant symptoms of intracranial compression (11). BM from NSCLC is highly heterogeneous and requires more specific molecular classification (12). However, the molecular pattern of BM from NSCLC are neglected, and more prognostic biomarkers of BM from NSCLC should be investigated for the precision classification and treatment.

WD repeat domain 5 (WDR5) is an essential component of histone methyltransferase complex SET1/MLL, which catalyzes the lysine 4 methylation of histone 3 (H3K4me) (13). H3K4me is one of the most important epigenetic modifications and its main function is to enhance the transcription and expression of the substrates, requiring the participation of WDR5 (14). Interestingly, WDR5 is reported to interact with both unmodified and methylated H3K4 *in vitro (* 15). The ectopic expression of WDR5 and negative correlations between WDR5 and prognosis are reported in several cancer types including prostate cancer, bladder cancer, cholangiocarcinoma, breast cancer and colorectal cancer (16–20). In lung cancer, WDR5 is reported to promote proliferation of lung adenocarcinoma by inducing SOX9 expression (21). In LC cell line A549 cells, WDR5 positively regulates P53 stability and arrests A549 cell cycle in G1 phase (22), which is conflicting with most studies indicating WDR5 as a tumor-promotor. However, the expression and clinical indications of WDR5 in BM from NSCLC are still unknown.

In our study, we established a cohort consisting of 70 patients with BM from NSCLC, and investigated the expression of WDR5 in the cohort. We analyzed the clinicopathological correlation of WDR5, and estimated WDR5 prognostic significance by univariate and multivariate analyses.

## Materials and methods

### Patient cohort and ethics

The inception cohort of our study consisted of the patients who underwent surgical resection of NSCLC, and were clinically diagnosed as asynchronous BM from NSCLC in the Second Hospital Affiliated to Shandong First Medical University and The First People’s Hospital of Tai’an from 2016 to 2020. The validation cohort was selected from the inception cohort following the criteria: (i) available formalin-fixed tumor tissues and follow-ups, (ii) no pre-operational adjuvant therapy including chemotherapy or radiotherapy, (iii) solitary and resectable BM lesion (iv) received gross total resection. Patients who died during the perioperative period were excluded from the study.

The study was approved by the Ethics Committee of the Second Affiliated Hospital of Shandong First Medical University and The First People’s Hospital of Tai’an. The written informed consent was obtained from each patient.

### Immunohistochemistry

Immunohistochemistry (IHC) was performed with streptavidin peroxidase complex method as previous described (23). The specimens were deparaffinized and rehydrated with xylene and graded alcohol first, followed by the antigen retrieval in boiled citrate buffer (pH = 6.0) for 30 minutes. After that, 3% hydrogen peroxide was applied to block the activity of endogenous peroxidase. 5% bovine serum albumin was administrated for diminishing the unspecific antigen binding. Primary antibody of WDR5 (R&D Systems, AF5810) was used to incubate the tissues at dilution of 1:50 overnight at 4°C, and the corresponding secondary antibody labelled with streptavidin-biotin-peroxidase reagent (Beyotime, Beijing, China) was applied to incubate the specimens for 30 minutes at room temperature. At last, the slides were incubated in the 3,3’-diaminobenzidine solution for visualization and tissues were counter-stained with haematoxylin.

### Evaluation of IHC

The semi-quantification method of IHC results was applied in our study. Two senior pathologists who were unaware of the clinical data estimated the IHC results and cases without consensus received a third pathologist for final determination. The IHC results were semi-quantified by IHC scores according to previous studies (24, 25), which were the multiplication product of score for staining intensity and positive cell percentage. The scores for staining intensity were identified as: score 0 for negative staining; score 1 for weak staining; score 2 for moderate staining and score 3 for strong staining. The scores of positively stained cell percentage were delineated as: score 1 for <25% positive cells; score 2 for 25%-50% positive cells; score 3 for 50%-75% positive cells; score 4 for 75%-100% positive cells. The final score ranged from 0 to 12, and the cut-off was identified by receiver operating characteristic (ROC) curve as previous studies (22, 26). Our cohort was separated to WDR5^low^ and WDR5^high^ subsets based on the cut-off of IHC score, which was defined as 3.0 in our study.

### Statistical analysis

All the statistical analyses were analyzed by SPSS 22.0 software (SPSS, Chicago, IL, USA). The correlations between WDR5 expression and clinicopathological factors were assessed by the chi-square test. The overall survival (OS) rates were calculated by Kaplan-Meier method and the statistical differences between subsets were assessed by the log-rank test. In multivariate analysis, cox-regression hazard model was applied to identify the independent prognostic factors. *P* value less than 0.05 was regarded as statistically significant.

## Results

### Basic information of the inception and validation cohort

The inception cohort consisted of 122 patients who were clinically diagnosed as asynchronous BM from NSCLC. There were 68 male and 54 female patients, with an average age as 61.4 years old. The validation cohort was selected from the inception cohort if patients received a surgical resection of BM. There were 70 patients in the validation cohort, comprised of 41 male patients and 29 female patients (**Table 1**). The average age of validation cohort was 62.7 years old, and the average survival time was 6.0 months. The basic line of inception and validation cohort had no significant difference in our study.

**Table 1:** Basic information of the inception and validation cohort.

Factors	Inception		Validation	
	number	percentage	number	percentage
Age				
≤60	45	36.89%	29	41.43%
>60	77	63.11%	41	58.57%
Gender				
Male	68	55.74%	41	58.57%
Female	54	44.26%	29	41.43%
Tumor size				
<3cm	44	36.07%	27	38.57%
≥3cm	78	63.93%	43	61.43%
Histological type				
Squamous carcinoma	67	54.92%	37	52.86%
Adenocarcinoma	55	45.08%	33	47.14%
KPS				
<80	50	40.98%	22	31.43%
≥80	72	59.02%	48	68.57%
WDR5				
Low	–	–	42	60.00%
High	-	-	28	40.00%

### WDR5 expression in brain metastasis of NSCLC

**Figure 1:**
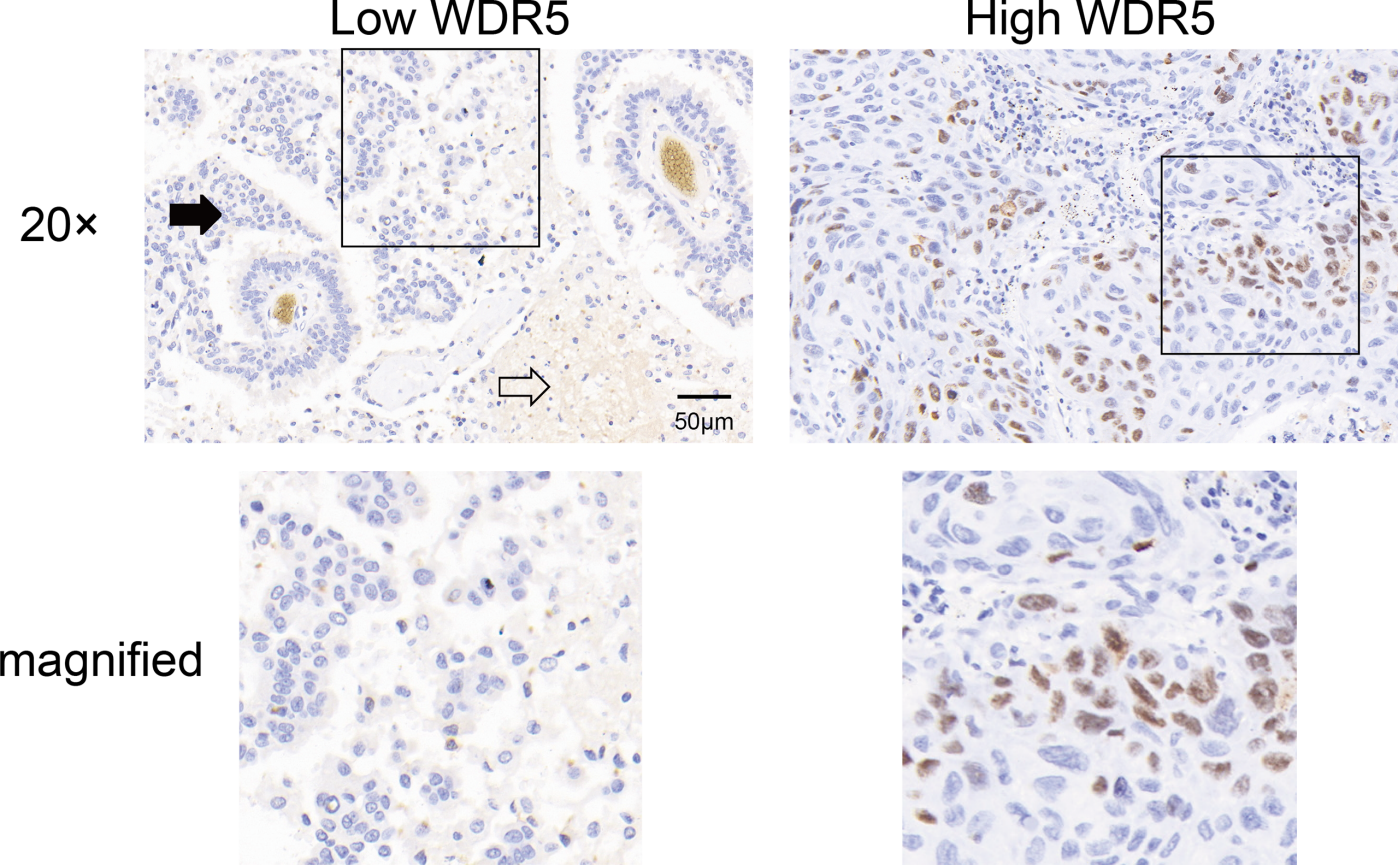
The expression of WDR5 in BM from NSCLC. The expression of WDR5 in BM from NSCLC was detected with IHC, dividing the cohort into high and low WDR5 expression. Black arrow indicates BM, and white arrow indicates brain tissue.

The expression and intracellular localization of WDR5 in BM of NSCLC were detected with IHC. WDR5 was mainly expressed in the nucleus of BMs (**Figure 1**). As described in *Materials and Methods* in detail, we categorized our cohort into subsets with low or high expression of WDR5. In our study, patients with low or high expression of WDR5 accounted for 60% and 40% respectively.

### Clinical relevance of WDR5 in BM from NSCLC

The correlations between WDR5 expression and clinicopathological factors were analyzed with the chi-square test (**Table 2**). The clinicopathological factors included the gender and age of patients, the tumor size, histological type, KPS score. In our study, no remarkable associations between WDR5 expression and above clinicopathological factors were observed.

**Table 2:** The correlation between WDR5 and the clinicopathological factors.

	WDR5		
Factors	Low	High	P*
Age			
≤60	17	12	0.843
>60	25	16	
Gender			
Male	25	16	0.843
Female	17	12	
Tumor size			
<3cm	18	9	0.367
≥3cm	24	19	
Histological type			
Squamous carcinoma	20	17	0.282
Adenocarcinoma	22	17	
KPS			
<80	11	11	0.248
≥80	31	17	

*Represents that data were analyzed with chi-square test.

### WDR5 predicted poor prognosis of BM from NSCLC

**Figure 2:**
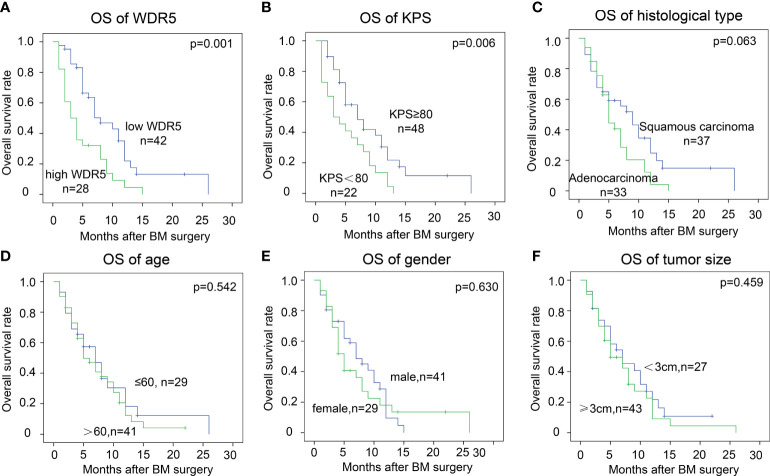
Prognostic significance of WDR5 and clinicopathological factors. The cohort was divided into subsets according to WDR5 expression and clinicopathological factors. High WDR5 **(A)** and low KPS **(B)** were significantly associated with low OS rate. Patients’ age **(C)**, gender **(D)**, tumor size **(E)** and histological type **(F)** had no statistically significant correlation with OS of BM.

Using log-rank method, we analyzed the prognostic significance of WDR5 and other clinicopathological factors (**Table 3**). High expression of WDR5 substantially correlated with poor prognosis of BM from NSCLC, suggesting that WDR5 was a prognostic biomarker (**Figure 2A**). In addition, high KPS was a favorable prognostic biomarker of BM from NSCLC (*P*=0.006) (**Figure 2B**). Interestingly, BMs from adenocarcinoma seemed to have a poorer prognosis compared with those from squamous carcinoma (**Figure 2C**). The 1-year OS rates of BM from squamous carcinoma and adenocarcinoma were 24.7% and 4.1%, respectively (**Table 3**). Other clinicopathological factors such as age, gender and tumor size, had no significant association with OS rates(**Figures 2D-F**).

**Table 3:** Prognostic significance in univariate and multivariate analyses.

Factors	1-year OS	P*	HR	95%CI	*P* ^#^
Age					
≤60	18.2	0.542	–	–	–
>60	12.3		–	–	
Gender					
Male	9.6	0.630	–	–	–
Female	18.1		-	-	
Tumor size					
<3cm	21.7	0.459	–	–	–
≥3cm	9.1		–	–	
Histological type					
Squamous carcinoma	24.7	0.063	1		
Adenocarcinoma	4.1		1.91	1.09-3.33	0.024
KPS					
<80	4.5	0.006	1		
≥80	21.8		2.23	1.26-3.94	0.006
WDR5					
Low	21.9	0.001	1		
High	4.6		2.48	1.43-4.31	0.001

*Represents that data were analyzed with log-rank test; ^#^represents that data were analyzed with Cox-regression hazard model.

By Cox-regression model, we further identified independent prognostic factors of BM from NSCLC (**Table 3**). The factors with *P* value less than 0.1 in univariate analysis were enrolled to the multivariate analysis, including WDR5 expression, KPS status and histological type of BM. All these factors were confirmed as the independently prognostic factors of BM. The odds of patients with high WDR5 of BM were 2.48 times of those with low WDR5 expression (*P*=0.001). More intriguingly, patients with BM from adenocarcinoma were more vulnerable to BM-related deaths than those with BM from squamous carcinoma.

## Discussion

More than half of BMs originate from lung cancer (27), and up to 50% of all LC suffer BM in different course of the LC (28). Although many progresses have been made in BM treatment such as whole brain irradiation, the prognosis of BM from NSCLC is still extremely dismal (29). Clinical management of BM from NSCLC is affected by many aspects such as the performance status and the overall health of the patient, meanwhile, the standard treatment of BM is still in exploration (6). Just like the primary tumor, BM is also very heterogeneous, but unfortunately, the molecular characters of BM from NSCLC are not well defined. Therefore, how to select the high-risk patients and apply the individual treatment is very important. Here we identified WDR5 as an independent prognostic biomarker of NSCLC-associated BM, indicating that patients with high WDR5 expression are much more high-risk to suffer BM and should receive more intense post-operational supervision.

Understanding the molecular mechanisms and screening potential biomarkers of NSCLC-associated BM may help discover more novel therapeutic drug targets to improve the prognosis of BM. Unfortunately, there is no effective method to predict the high-risk patients who are susceptible to BM. Several studies attempted to select high-risk patients of BM. For example, historical data from 1973 to 2011 using the SEER database showed that 9% NSCLC patients without previously non-metastasis would suffer BM during the disease course, whereas this number was as high as 18% to patients who suffer small cell lung cancer patients without previous metastasis (30). In general, age of ≤ 60 years, non-squamous cell carcinoma and the presence of clinical bulky mediastinal lymph nodes (> 2 cm) are reported to be correlated with a high BM rate in locally advanced NSCLC (31–34). However, these results have not got total consensus, and most studies are focused on the clinicopathological factors but not the molecular features. Consistent with previous studies (35, 36), we demonstrated that low KPS and adenocarcinoma are associated with more unfavorable prognoses in our study. Although the sample size of our cohort of BM from NSCLC is not small (n=70), more multi-center and perspective cohort studies should be conducted to identify more effective biomarkers and possible drug targets of BM.

The oncogenic role of WDR5 has not been well elucidated. Most studies focused on the function of WDR5 as a component of histone methyltransferase complex. Emerging evidence gradually revealed the precise regulatory mechanism of WDR5 on H3K4me. For example, the phosphorylation of WDR5 is also shown to modulate the WDR5 function and thus regulate H3K4me of substrates (37). Moreover, more evidence showed that WDR5 can promote gene transcription in addition to modulating H3K4me, such as binding with c-Myc and thus regulating transcription (38). Here we identified WDR5 as an independent biomarker indicating the unfavorable prognosis of BM from NSCLC, but underlying molecular mechanism was not investigated here. Moreover, whether WDR5 overexpression results in easier BM is also an interesting and worthy topic to investigate because there is still no effective biomarker to predict BM risk to date. Several WDR5 inhibitors are in clinical trial for the treatment of acute myeloid leukemia. Our results also indicate that WDR5 may be a potential drug target of BM from NSCLC, and targeting WDR5 may be a possible treatment option.

In conclusion, we established a cohort of patients with asynchronous BM from NSCLC, and investigated the expression of WDR5 for the first time. Interestingly, high expression of WDR5 can predict the poor prognosis of BM, and WDR5 is an independent prognostic biomarker of BM from NSCLC. Our results indicate that patients with WDR5 overexpression are more high-risk to suffer BM-related death, and should receive stricter post-operational supervision. Our study provides more evidence for the molecular classification of BM from NSCLC, and may help guide the individual treatment of BM.

## Data availability statement

The original contributions presented in the study are included in the article/supplementary material. Further inquiries can be directed to the corresponding author.

## Ethics statement

The study was approved by the Ethics Committee of the Second Affiliated Hospital of Shandong First Medical University and The First People’s Hospital of Tai’an. The written informed consent was obtained from each patient. The patients/participants provided their written informed consent to participate in this study.

## Author contributions

ZL, NL, NW and YJ collected the specimen and established the cohort. ZL and NL performed the experiments and analyzed the data. CT designed the study and wrote the paper. All authors contributed to the article and approved the submitted version.

## Conflict of interest

The authors declare that the research was conducted in the absence of any commercial or financial relationships that could be construed as a potential conflict of interest.

## Publisher’s note

All claims expressed in this article are solely those of the authors and do not necessarily represent those of their affiliated organizations, or those of the publisher, the editors and the reviewers. Any product that may be evaluated in this article, or claim that may be made by its manufacturer, is not guaranteed or endorsed by the publisher.
